# FGF18–FGFR2 signaling triggers the activation of c-Jun–YAP1 axis to promote carcinogenesis in a subgroup of gastric cancer patients and indicates translational potential

**DOI:** 10.1038/s41388-020-01458-x

**Published:** 2020-09-15

**Authors:** Jinglin Zhang, Chi Chun Wong, Kam Tong Leung, Feng Wu, Yuhang Zhou, Joanna H. M. Tong, Ronald C. K. Chan, Hui Li, Yifei Wang, Huan Yan, Liping Liu, William K. K. Wu, Michael W. Y. Chan, Alfred S. L. Cheng, Jun Yu, Nathalie Wong, Kwok Wai Lo, Ka Fai To, Wei Kang

**Affiliations:** 1 Department of Anatomical and Cellular Pathology, State Key Laboratory of Translational Oncology, Prince of Wales Hospital, The Chinese University of Hong Kong, Hong Kong SAR, PR China; 2 Institute of Digestive Disease, State Key Laboratory of Digestive Disease, The Chinese University of Hong Kong, Hong Kong SAR, PR China; 3 Li Ka Shing Institute of Health Science, Sir Y.K. Pao Cancer Center, The Chinese University of Hong Kong, Hong Kong SAR, PR China; 4 Department of Pediatrics, The Chinese University of Hong Kong, Hong Kong SAR, PR China; 5 Department of Hepatobiliary and Pancreatic Surgery, Shenzhen People’s Hospital, Second Clinical Medical College of Jinan University, Shenzhen, Guangdong Province PR China; 6 Department of Anaesthesia and Intensive Care, The Chinese University of Hong Kong, Hong Kong SAR, PR China; 7 Department of Life Science, National Chung Cheng University, Chiayi, Taiwan; 8 School of Biomedical Sciences, The Chinese University of Hong Kong, Hong Kong SAR, PR China; 9 Department of Medicine and Therapeutics, The Chinese University of Hong Kong, Hong Kong SAR, PR China; 10 Department of Surgery, The Chinese University of Hong Kong, Hong Kong SAR, PR China

**Keywords:** Gastric cancer, Tumour biomarkers, Growth factor signalling

## Abstract

Fibroblast growth factor receptor type 2 (FGFR2) has emerged as a key oncogenic factor that regulates gastric cancer (GC) progression, but the underlying mechanism of FGF–FGFR2 signaling pathway remains largely unknown. To identify the potential molecular mechanisms of the oncogenic FGFR2 in gastric carcinogenesis and convey a novel therapeutic strategy, we profiled the FGFR alterations and analyzed their clinical associations in TCGA and Hong Kong GC cohorts. We found that FGFR2 overexpression in GC cell lines and primary tumors predicted poor survival and was associated with advanced stages of GC. Functionally, growth abilities and cell cycle progression of GC were inhibited by inactivation of ERK–MAPK signal transduction after FGFR2 knockdown, while apoptosis was promoted. Meanwhile, the first-line anti-cancer drug sensitivity was enhanced. RNA-seq analysis further revealed that YAP1 signaling serves as a significant downstream modulator and mediates the oncogenic signaling of FGFR2. When stimulating FGFR2 by rhFGF18, we observed intensified F-actin, nuclear accumulation of YAP1, and overexpression of YAP1 targets, but these effects were attenuated by either FGFR2 depletion or AZD4547 administration. Additionally, the FGF18–FGFR2 signaling upregulated YAP1 expression through activating c-Jun, an effector of MAPK signaling. In our cohort, 28.94% of GC cases were characterized as FGFR2, c-Jun, and YAP1 co-positive and demonstrated worse clinical outcomes. Remarkably, we also found that co-targeting FGFR2 and YAP1 by AZD4547 and Verteporfin synergistically enhanced the antitumor effects in vitro and in vivo. In conclusion, we have identified the oncogenic FGF–FGFR2 regulates YAP1 signaling in GC. The findings also highlight the translational potential of FGFR2–c-Jun–YAP1 axis, which may serve as a prognostic biomarker and therapeutic target for GC.

## Introduction

Gastric cancer (GC) is one of the most lethal malignancies globally and remains a prevalent malignancy in Eastern Asia areas, including Hong Kong [1]. Recently, the number of GC cases is increasing among the younger population [2–4]. Regarding the histological characteristics, over 90% of the cases are adenocarcinomas. By Lauran’s classification, GC can be subgrouped as the intestinal type and diffuse type [5]. A more comprehensive molecular classification has been proposed by The Cancer Genome Atlas (TCGA) consortium, consisting of four major molecular subtypes: microsatellite instability, Epstein-Barr virus-associated GC, chromosomal instability, and genomically stable (GS) [6].

GC is a heterogeneous disease with multiple pathogenic mechanisms such as aberrant crosstalk of signaling pathways [7–9]. As we previously reported, a prominent fibroblast growth factor (FGF) family member FGF18 is highly upregulated in GC. We found FGF18 stimulation activates several signaling pathways in GC, such as ERK–MAPK and TGFβ–SMAD2/3 [10]. The FGF receptors (FGFRs) are known as transmembrane tyrosine kinases. Activation of FGFR initiates the downstream cascades by binding with certain FGFs [11]. Aberrant activation of FGFRs contributes to tumorigenesis by stimulating cell proliferation and migration [12, 13]. In cancer cells, genetic and epigenetic modifications of FGFR2 lead to overexpression, fusion protein, and increased ligand-binding affinity. Meanwhile, somatic mutations on the functional domains of FGFRs can trigger constitutive self-activation [12, 14, 15]. Preclinical and experimental work has validated the tumor-promoting role of FGFR2 in tumorigenesis and metastasis of GC [16–19]. *FGFR2* gene amplification rates about 5–10% in GC cases [20]. Besides, alternative splicing produced FGFR2 isoforms, FGFR2b and FGFR2c, harbor exclusive binding affinities to FGFs. FGFR2b overexpression is known as an accurate indicator for GC patients who are more sensitive to FGFR2 inhibitors [21].

Targeting aberrant activation of FGF–FGFR represents a novel therapeutic strategy in GC. Specific antibodies or small molecules targeting the FGF–FGFR have been developed and are undergoing clinical trials [22]. However, tyrosine kinase inhibitors are effective only in a small portion of GC patients. High intra-tumor heterogeneity is one of the causes of drug resistance [23], while other resistant mechanisms remain poorly understood. In several selective pan-FGFR inhibitors, AZD4547 demonstrates the most promising effect in a subgroup of patients with FGFR2 amplification [24]. Nevertheless, FGFR-targeted treatments are still under evaluation in Phase I/II clinical trials.

In this study, we aim to elucidate the molecular mechanisms and evaluate the translational potential of FGF–FGFR signaling cascade as a biomarker and therapeutic target in GC.

## Results

### FGFR2 is overexpressed and predicts poor survival of GC patients

**Fig. 1:**
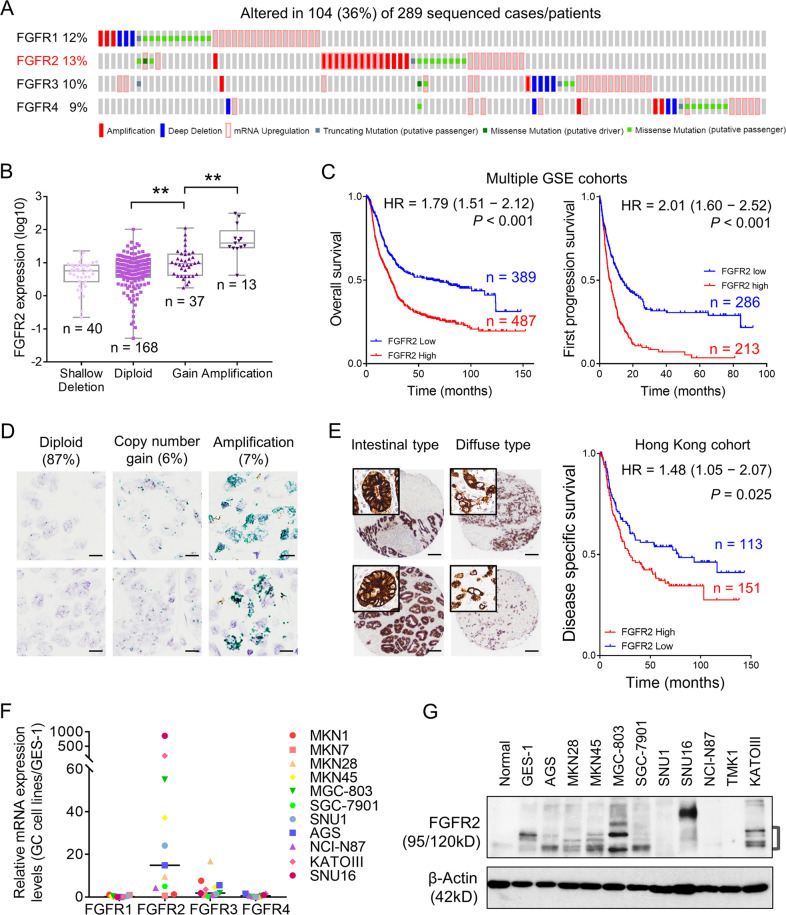
FGFR2 is upregulated in GC and serves as a poor prognostic biomarker. **a** Genetic alterations (gene amplification, deep deletion, or somatic mutation) or mRNA upregulation of the main FGFR members in primary samples from the TCGA-GC cohort (*n* = 104, total alteration rate: 36%). **b** Correlation analysis of FGFR2 copy number changes and its mRNA expression in TCGA-GC samples (*n* = 258, **p < 0.001). **c** Upregulated FGFR2 mRNA expression correlates with shorter overall and first progression survival in multiple GSE cohorts (GSE14210, GSE15459, GSE22377, GSE29272, GSE51105, and GSE62254) (*p* < 0.001; HR, hazard ratio). **d** CISH analysis of FGFR2 (green) for DNA aberration detection in primary GC samples (Hong Kong cohort, *n* = 264; scale bar, 20 μm). Cases with copy number gain and cases with gene amplification count for 6% and 7%, respectively. **e** Representative images of IHC staining of FGFR2 in GC tissue microarray (TMA). FGFR2 predominantly localized in the cytoplasmic and membrane of diffuse/intestinal type cancer cells (scale bar, 100 μm for lower resolution and 20 μm for higher resolution). Overexpressed FGFR2 is associated with poor disease-specific survival in primary GCs (Hong Kong cohort, *n* = 264; *p* = 0.025; HR = 1.48). **f** The mRNA expression of FGFR1-4 in 11 GC cell lines. **g** FGFR2 protein expression in GC cell lines and normal gastric epithelial samples.

By the cBioportal analysis, TCGA dataset was used to demonstrate the genetic and epigenetic alterations of FGFRs in primary GC cases. Thirty-six percent of the cases have at least one alteration in FGFR members (Fig. 1a), including copy number changes, somatic mutations, and mRNA upregulation. Genetic or mRNA change in *FGFR1-4* accounts for 12%, 13%, 10%, and 9% of GC cases, respectively, and FGFR2 expression demonstrates a positive correlation with its copy number gain/gene amplification, suggesting that copy number aberration partially contributes to overexpression of FGFR2 (*p* < 0.001, Fig. 1b). By Kaplan–Meier plotter analysis (based on multiple GSE cohorts: GSE14210, GSE15459, GSE22377, GSE29272, GSE51105, and GSE62254), high expression of FGFR2 was associated with poor overall survival and first progression survival (Fig. 1c). Moreover, the copy number aberrations of *FGFR2* were assessed by using FGFR2 probes for CISH analysis in 264 samples of our in-house Hong Kong cohort. Eighty-seven percent of cases were counted to be diploid, 6% possessed copy number gain, and the rest 7% of cases harbored gene amplification (Fig. 1d). At the protein level, FGFR2 predominantly localized in the cell cytoplasm and membrane both in intestinal and diffuse type GC by IHC analysis (left panel, Fig. 1e) and can be categorized into low and high expression groups (total 264 cases were counted for scoring) according to our previous method [25]. Significantly, overexpression of FGFR2 indicated a poor disease-specific survival of patients (right panel, Fig. 1e) and correlated with several clinicopathologic parameters, implying aberrant FGFR2 activation might contribute to advanced stage of GC. By univariate Cox regression analysis, older age, diffuse histological type, high grade, advanced stage, lymph node metastasis, and FGFR2 overexpression were correlated with unfavorable outcomes (Supplementary Table S1). By multivariate analysis, only older age and advanced stage were independently associated with poor prognosis (Supplementary Table S2). Further, the mRNA expression of four FGFRs was evaluated in eleven GC cell lines. FGFR2 showed dramatic upregulation in most of these GC cell lines compared with the normal gastric cell line GES-1, while other FGFR members did not demonstrate such significant upregulation (Fig. 1f). Upregulating protein level of FGFR2 was consistent with its mRNA upregulation in most of the GC cell lines, especially in KATO III, MGC-803, AGS, and MKN28 cells (Fig. 1g).

### FGFR2 knockdown exerts an antineoplastic effect in GC

**Fig. 2:**
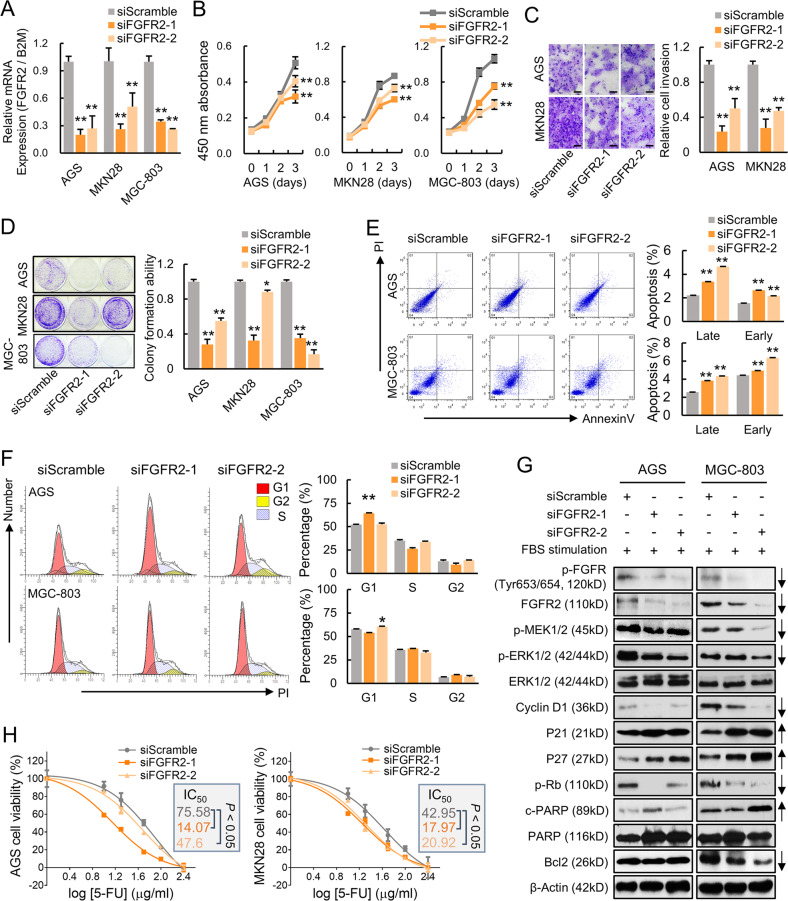
Depletion of FGFR2 in GC cells exhibits an antitumor effect. **a** The mRNA expression of FGFR2 in AGS, MKN28, and MGC-803 cells after transfection with siFGFR2s (**p < 0.001). **b** Knockdown of FGFR2 suppressed cell proliferation of the cancer cells (**p < 0.001). **c** siRNA-mediated knockdown of FGFR2 inhibited monolayer colony formation ability of GC cells (*p < 0.05; **p < 0.001). **d** Knocking down FGFR2 decreased cell invasion ability (**p < 0.001) (Scale bar, 50 μm). **e** siFGFR2 induced late and early apoptosis which was manifested by flow cytometry analysis (**p < 0.001). **f** Cell cycle distribution of siFGFR2 transfectants and siScramble control group by flow cytometry analysis. **g** Western blot analysis of MARK signaling, cell cycle regulators, and apoptosis biomarker cleaved PARP after FGFR2 knockdown. **h** The antitumor efficiency of 5-FU after FGFR2 knockdown in AGS and MKN28 was demonstrated by IC_50_. All experiments were performed in triplicate.

To further elucidate the functional role of FGFR2, siRNA-mediated knockdown was performed. Two independent commercial siRNAs targeting FGFR2 and one control siScramble were transfected into several GC cell lines (AGS, MKN28, MGC-803) with high level of FGFR2 accordingly. Knockdown efficiency was firstly confirmed by qRT-PCR (Fig. 2a). FGFR2 knockdown induced significant inhibition of cell proliferation, monolayer colony formation ability, and cell invasion ability in AGS, MKN28, and MGC-803 (Fig. 2b–d). To further investigate the antitumor effect of FGFR2 silencing, we employed flow cytometry to analyze apoptosis and cell cycle distribution. As indicated, the proportion of both early and late stage apoptotic cells were significantly increased in siFGFR2 transfectants (Fig. 2e). As to cell cycle distribution, G1 phase population was increased in siFGFR2 transfectants (Fig. 2f). Looking at the alteration of apoptotic and cell cycle markers, cleaved PARP was increased, cell cycle related proteins cyclin D1 and p-Rb were inactivated, and p21 and p27 expression were induced in siFGFR2 transfectants compared with siScramble control. Moreover, FGFR2 depletion inactivated the ERK–MAPK signaling (Fig. 2g). To assess whether FGFR2 related to first-line anti-cancer drug resistance in GC, cells with either siFGFR2 or siScramble control were treated with escalating concentrations of fluorouracil (5-FU) and cell viability was detected subsequently. IC_50_ was significantly decreased by the transfection of both FGFR2 siRNAs compared with siScramble control (Fig. 2h). Together, we observed that knockdown of FGFR2 not only triggered an antineoplastic effect by inducing apoptosis and inhibiting cell cycle progression, but also enhancing the 5-FU sensitivity in GC cells.

### YAP1 is a prominent downstream of FGF–FGFR2 signaling

**Fig. 3:**
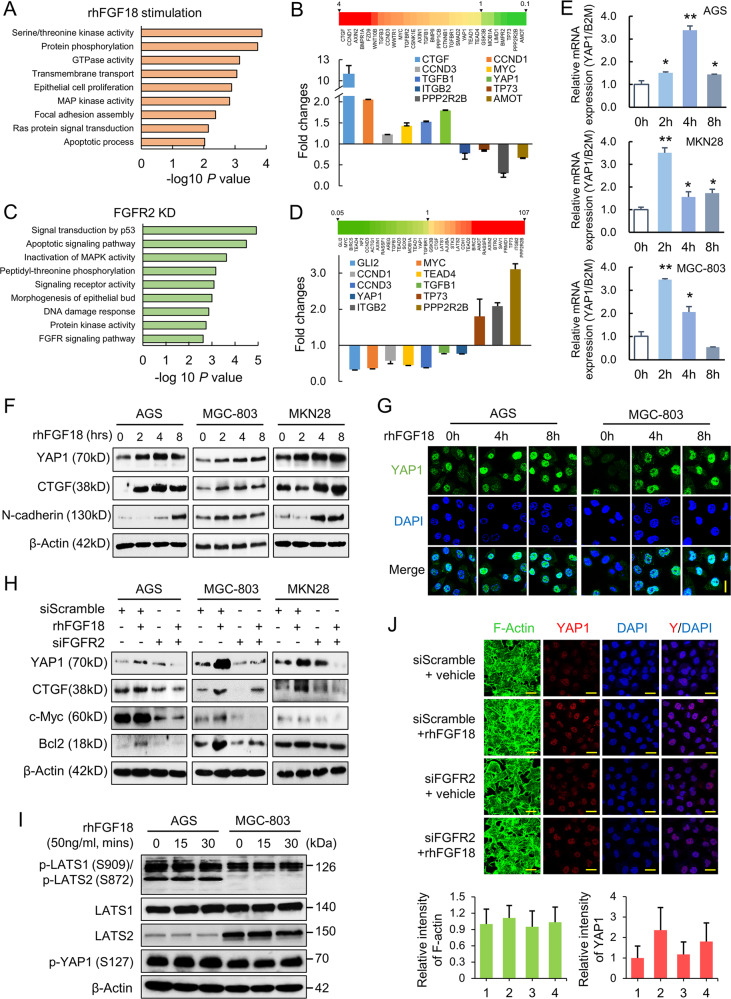
FGF18–FGFR2 activate YAP1 expression. **a** The top-ranking geneset enrichments by gene ontology (GO) analysis after rhFGF18 (50 ng/ml) stimulation in MGC-803 cells. **b** The heatmap demonstrates the differentially expressed genes in the Hippo-YAP1 pathway revealed by RNA-seq. Expressions of crucial genes were validated by qRT-PCR. **c** The top altered geneset enrichments by GO analysis after FGFR2 knockdown in MGC-803 cells. **d** The differentially expressed Hippo-YAP1 genes in the siFGFR2 transfectants. Suppression of YAP1 and related genes were validated by qRT-PCR. **e** rhFGF18 (50 ng/ml) increased YAP1 mRNA expression in a time-dependent manner (*p < 0.05; **p < 0.001). **f** Western blot analysis indicated the upregulation of YAP1 together with CTGF and N-cadherin after rhFGF18 incubation (50 ng/ml) in all the three cell lines. **g** Immunofluorescence staining validated the upregulation of YAP1 in AGS and MGC-803 after rhFGF18 stimulation (50 ng/ml; scale bar, 20 μm). **h** The stimulatory effect induced by rhFGF18 was abolished by FGFR2 knockdown. **i** The western blot analysis of the upstream components in Hippo-YAP1 pathway. p-LATS1/2 (S909/S872) and p-YAP1 (S127) did not show observable changes after rhFGF18 stimulation. **j** Tensioned F-actin (green) rearrangement and YAP1 nuclear translocation (red) is triggered by rhFGF18 stimulation (50 ng/ml) but abolished by knocking down FGFR2 in MGC-803 (scale bar, 20 μm). In the statistical bar chart, 1 to 4 represent siScramble + vehicle, siScramble + rhFGF18, siFGFR2 + vehicle, and siFGFR2 + rhFGF18, respectively.

We previously identified FGF18 as a potent oncogenic driver in gastric tumorigenesis [10]. As FGFR2 highly expressed in GC cells, we hypothesized that FGFR2 is the major responding receptor for FGF18 in GC. To investigate the functional downstream of FGFR2, we performed RNA-seq on MGC-803 cells, which either treated with recombinant human FGF18 (rhFGF18) or transfected with a siFGFR2. In rhFGF18-stimulated cells, Gene Ontology (GO) analysis indicated that the deregulated genes were mainly enriched in biological processes including serine/threonine kinase activity, protein phosphorylation, and GTPase activity (Fig. 3a and Supplementary Table S3). Among the top-ranking signaling pathways, we observed that multiple genes related to Hippo-YAP1 signaling were highly altered. Expression levels of these genes were further validated by qRT-PCR. YAP1, MYC, CTGF, and CCND1 were upregulated, while TP73 and AMOT were decreased (Fig. 3b and Supplementary Table S3). Meanwhile, FGFR2 knockdown was highly associated with the signal transduction of p53, apoptotic signaling pathway, and inactivation of MAPK activity (Fig. 3c and Supplementary Table S3). Most genes validated for rhFGF18 stimulation were also highly altered by FGFR2 knockdown. The YAP1–TEAD4 complex and its downstream MYC, CCND1, and CCND3 were downregulated, whereas the apoptosis-related genes, TP73 and PPP2R2B were upregulated (Fig. 3d and Supplementary Table S3). YAP1 is found as a prominent downstream factor of the FGF18–FGFR2 signaling. To elucidate the potential role of YAP1 in this signaling, rhFGF18 was used to simulate the FBS-deprived GC cells for different time points (0–8 h). At the mRNA level, YAP1 was upregulated in GC cell lines after 2 and 4 h of stimulation (Fig. 3e). At the protein level, YAP1, CTGF, and a mesenchymal marker N-cadherin were elevated after rhFGF18 stimulation in a time-dependent manner (Fig. 3f). We also employed immunofluorescence staining to confirm the upregulation of YAP1. Nuclear YAP1, the functional oncogenic form of YAP1, was remarkably enriched in nuclei after 4 and 8 h of stimulation (Fig. 3g). We further identified whether FGFR2 knockdown abolishes the activation effect of rhFGF18. With FGFR2 depletion, YAP1, CTGF, c-Myc, and Bcl2 were no longer responding to the rhFGF18 stimulation in AGS, MKN28, and MGC-803 cells, indicating that the FGF18 activated YAP1 signaling is suppressed (Fig. 3h). To confirm whether the Hippo signaling pathway has crosstalk with the FGF18–FGFR2 signaling, the upstream regulators of YAP1 were examined. Phospho-LATS1/2 (S909/S872), LATS1, LATS2, and phosphor-YAP1 (S127) did not show significant changes in the rhFGF18-stimulated cells, hinting that rhFGF18 activated YAP1 expression is Hippo-independent (Fig. 3i). Previous evidence indicated that the nuclear activation of YAP1 is intermediated by the dynamics of F-actin cytoskeleton [26]. Apart from the YAP1 translocation, rhFGF18 also altered the arrangement of F-actin cytoskeleton. siFGFR2 transfection abolished YAP1 nuclear translocation and ameliorated the F-actin cytoskeleton to the naïve status even with rhFGF18 stimulation (Fig. 3j).

### AZD4547 suppresses YAP1 signaling through inhibiting FGFR2

**Fig. 4:**
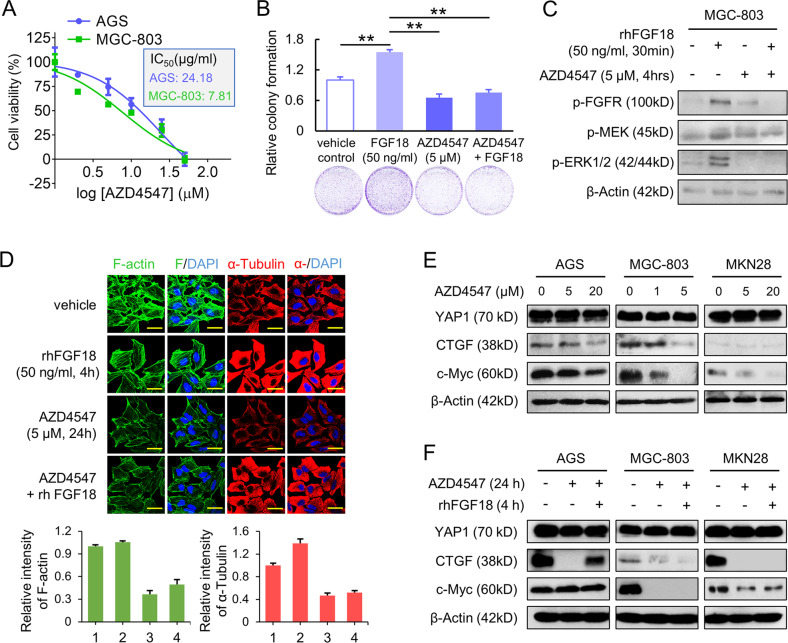
AZD4547 exerts tumor suppressive role by inhibiting FGFR2 and YAP1 signaling. **a** The IC_50_ value of AZD4547 in AGS and MGC-803 cells. **b** Monoclonal colony formation of MGC-803 cells under the treatment of rhFGF18 (50 ng/ml), AZD4547 (5 µM), or AZD4547/rhFGF18, respectively (**p < 0.001). **c** FGFR and the MEK–ERK signaling were activated by the rhFGF18 stimulation (50 ng/ml) but quenched by AZD4547 (5 µM). **d** The intensity of migration related F-actin (green) and cell division-related α-Tubulin (red) was analyzed in MGC-803 cells by immunofluorescence staining (scale bar, 20 μm). In the statistical bar chart, 1 to 4 represent vehicle, rhFGF18, AZD4547, and rhFGF18 + AZD4547, respectively. **e** AZD4547 dose-dependently decreased the downstream targets of YAP1, including CTGF and c-Myc (24 h). **f** rhFGF18 (50 ng/ml) partly restored CTGF and c-Myc expression in AZD4547 (25 µM for AGS, 5 µM for MGC-803, 25 µM for MKN28) treated cell lines.

To explore whether inhibition of FGFR2 affects the potential downstream YAP1 signaling in GC, AGS and MGC-803 were treated with a pan-FGFR inhibitor AZD4547, which is highly sensitive to FGFR2. According to the IC_50_, MGC-803 cells are more sensitive to AZD4547 than AGS (Fig. 4a). The monoclonal colony formation ability of MGC-803 cells was increased by rhFGF18 (50 ng/ml), while AZD4547 (5 μM) abrogated the effect (Fig. 4b). Treatment of rhFGF18 increased the level of phosphorylated FGFR (p-FGFR), MEK (p-MEK), and ERK (p-ERK), but additional AZD4547 abolished the FGFR and MEK–ERK activation (Fig. 4c). It not only indicated that rhFGF18 activates FGFR2, but also confirmed that the MAPK signaling is responsible for signal transduction of the FGF18–FGFR2 axis. Furthermore, inhibition of FGFR2 by AZD4547 altered the cytoskeleton structure in MGC-803 cells. F-actin associates with cell migration, and α-Tubulin is an indispensable factor for cell division. With FGF18 stimulation, both F-actin and α-Tubulin were intensified. While in the cells treated with AZD4547, F-actin and α-Tubulin were disrupted. The stimulatory effect of rhFGF18 did not fully restore the effects caused by AZD4547 (Fig. 4d). Under treatment of AZD4547, the YAP1 downstream effectors, CTGF and c-Myc, were suppressed in a dose-dependent manner (Fig. 4e). CTGF and c-Myc were only partly rescued by rhFGF18 stimulation in GC cells, compared with those by AZD4547 treatment only (Fig. 4f). It indicated that FGF18–FGFR2 signaling contributes to the oncogenic function of YAP1 and its downstream targets. We transfected KATO III, a cell line with high *FGFR2* amplification, with siFGFR2 (Supplementary Fig. S1A). Knocking down FGFR2 significantly impaired the cell proliferation ability in KATO III (Supplementary Fig. S1B). We also treated KATO III-derived tumors with AZD4547. Tumor growth was significantly retarded compared with vehicle control (Supplementary Fig. S1C). Additionally, expression of FGFR2 was reduced in KATO III-derived xenografts after AZD4547 treatment. Expression of YAP1 and Ki-67 were also decreased, but cleaved-caspase-3 was increased (Supplementary Fig. S1D). Together, targeting FGFR2 by small molecules inhibited YAP1 expression, suppressed tumor growth, and enhanced apoptosis in GC.

### FGF18–FGFR2 regulate YAP1 expression through c-Jun activation

**Fig. 5:**
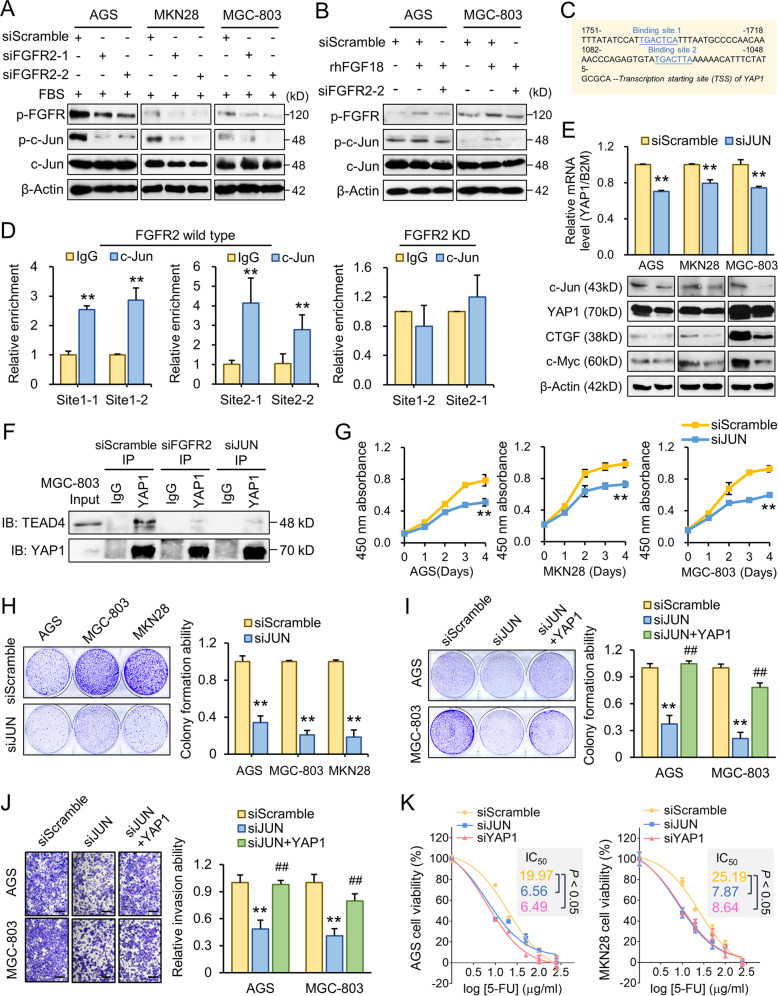
FGF18–FGFR2 regulates YAP1 expression through c-Jun. **a** FGFR2 knockdown inhibited FGFR and c-Jun phosphorylation in GC cells. **b** rhFGF18 stimulation promoted FGFR and c-Jun phosphorylation, but the effect was attenuated by FGFR2 depletion. **c** Two putative binding sites of c-Jun on the YAP1 promoter region (2000 bp upstream of YAP1 transcription starting site). **d** ChIP-qPCR analysis revealed the enrichment of c-Jun on YAP1 promoter in MGC-803 cells. IgG was applied as negative control and data normalized by input (**p < 0.001). c-Jun enrichment abolished by FGFR2 knockdown//. **e** The knockdown of JUN decreased mRNA and protein expression of YAP1 in GC cells (**p < 0.001) as well as the YAP1 downstream targets, CTGF, and c-Myc. **f** Immunoprecipitation of the YAP1/TEAD4 complex was determined by western bot in MGC-803 with siScramble, siFGFR2, or siJUN transfection, respectively. IgG was applied as a negative control. **g** GC cell proliferation was inhibited by JUN knockdown (**p < 0.001). **h** The impaired monolayer colony formation by JUN depletion (**p < 0.001). **i** YAP1 re-expression rescued the impaired monolayer colony formation caused by JUN knockdown in AGS and MGC-803 cells (**, siJUN vs. siScramble, *p* < 0.001; ##, siJUN vs. siJUN + YAP1, *p* < 0.001). **j** Cell invasive ability was suppressed by JUN knockdown but reversed by ectopic expression of YAP1 (**, siJUN vs. siScramble, *p* < 0.001; ##, siJUN vs. siJUN + YAP1, *p* < 0.001) (scale bar, 50 μm). **k** siJUN and siYAP1 increased the first-line anti-cancer drug (5-FU) sensitivity. IC_50_ was shown accordingly.

Although YAP1 is known as a proto-oncogene in multiple cancers, its transcriptional regulation remains largely unknown. To identify the mediator between FGFR2 and YAP1, we searched the JASPAR 2020 database [27] for core transcription factors (TFs) and noticed JUN is frequently shown among the high-ranked TFs enriched in the YAP1 promoter region (Supplementary Table S4). c-Jun has been well characterized as a downstream effector of the MAPK signaling pathway. We hypothesized that c-Jun is a potential TF underlying FGFR2-MAPK signaling and regulates YAP1 expression. GC cell lines were transfected with siFGFR2s to yield a knockdown effect. With serum deprivation and a followed FBS stimulation, p-FGFR and p-c-Jun were reduced in all three GC cell lines transfected with siFGFR2 (Fig. 5a). rhFGF18 stimulation activates p-FGFR and p-c-Jun. However, the stimulatory effect was eliminated in the siFGFR2 transfectants. It further implies the FGF18–FGFR2 activation promotes c-Jun phosphorylation (Fig. 5b). By TRANSFAC (version 8.3) prediction, two c-Jun binding sites were identified and selected within 2000 bp upstream from the transcription start site of *YAP1* (Fig. 5c). The enrichment of c-Jun on the *YAP1* promoter region was confirmed by ChIP-qPCR. When knocking down FGFR2, the enrichment of c-Jun was inhibited, suggesting that FGFR2 is required for the transcription regulation of c-Jun on *YAP1* (Fig. 5d). siRNA-mediated JUN knockdown significantly decreased YAP1 mRNA expression. Consistently, the protein level of YAP1 together with its downstream factors, CTGF and c-Myc, were downregulated by siJUN (*p* < 0.001, Fig. 5e). Since YAP1 regulates multiple oncogenes during gastric carcinogenesis by cooperating with TEADs, the interaction status of YAP1/TEAD4 was examined by immunoprecipitation assay in MGC-803 cells. Knocking down either FGFR2 or JUN dissociated the YAP1/TEAD4 complex (Fig. 5f). The knockdown of JUN led to the suppression of cell proliferation (Fig. 5g) and monolayer colony formation in GC cell lines (Fig. 5h). Further, ectopic overexpression of YAP1 in siJUN transfectants (Supplementary Fig. S1E) partly rescued the suppressed colony formation (Fig. 5i) and cell invasion abilities (*p* < 0.001, Fig. 5j). These results support that c-Jun mediated the regulation of FGF18–FGFR2 on YAP1 expression. To assess the therapeutic potential of targeting JUN and YAP1, a first-line chemo-drug 5-FU was employed to treat the siJUN, siYAP, or siScramble transfectants, respectively. IC_50_ was decreased in both siJUN and siYAP1 transfectants (Fig. 5k), indicating the JUN and YAP1 might be involved in the chemoresistance of GC.

### Co-overexpression of FGFR2–c-Jun–YAP1 axis indicates poor outcomes

**Fig. 6:**
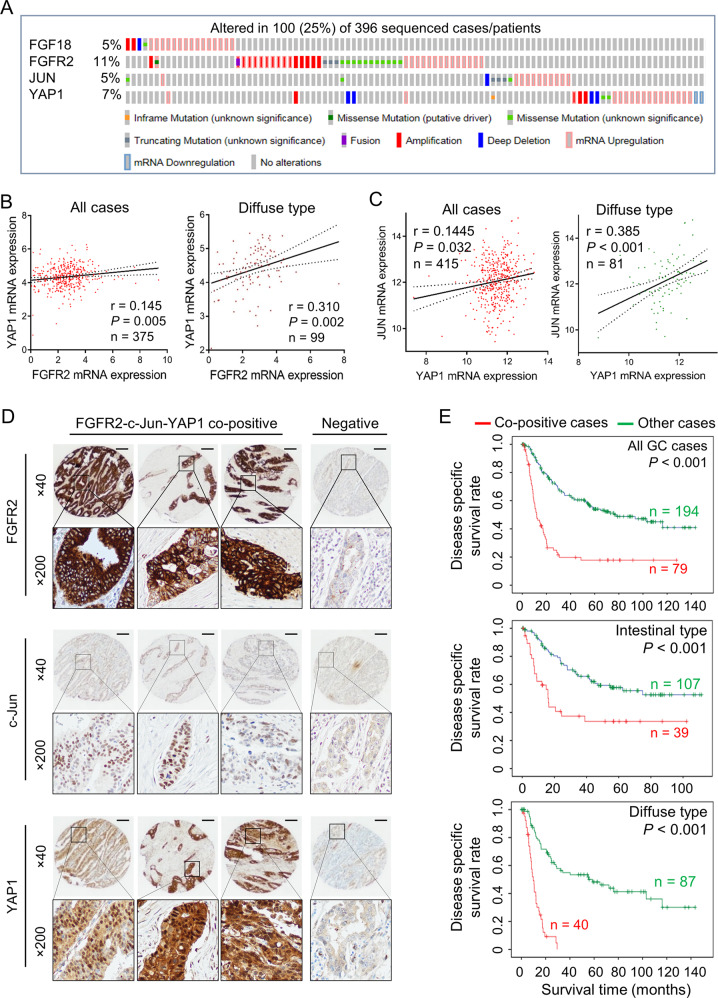
The co-activation FGFR2–c-Jun–YAP1 axis correlates with unfavorable outcomes. **a** The genetic aberration and mRNA expression alterations of FGF18, FGFR2, JUN, and YAP1 in the TCGA cohort. The changes between each gene were mutually exclusive (*p* < 0.001). **b** The expression correlation of FGFR2 and YAP1 in primary GC cases and diffuse type GC cases (TCGA cohort). **c** The correlation analysis of YAP1 and JUN in primary GC samples (TCGA cohort). In diffuse type GC cases, the positive correlation is more stringent (*p* < 0.001). **d** Representative IHC images of FGFR2–c-Jun–YAP1 co-positive and negative cases. The primary samples were stratified as two subgroups according to the expression of membrane/cytoplasmic FGFR2, nuclear c-Jun, and nuclear YAP1 accumulation (scale bar, 20 μm). **e** GC cases with FGFR2–c-Jun–YAP1 co-activation were associated with poor disease-specific survival (*p* < 0.001). In diffuse type GC, the co-overexpression of these three biomarkers predicted more unfavorable comes (*p* < 0.001).

In the TCGA-GC cohort, genetic and epigenetic alterations of the FGF18–FGFR2–JUN–YAP1 axis were profiled by the cBioportal. Changes of each gene were mutually exclusive (*p* < 0.001), implying a single component change in this axis might drive gastric tumorigenesis (Fig. 6a). A positive correlation between FGFR2 and YAP1 expression was observed in GC cases. Herein, the diffuse type cases display a more stringent correlation (Fig. 6b). A positive correlation between YAP1 and JUN expression was also observed. The expression correlation is much stronger in the diffuse type GC samples (Fig. 6c). We, therefore, proposed that FGFR2–c-Jun–YAP1 was co-activated in a proportion of primary GC cases. In a Hong Kong cohort, tissue microarray samples of GC were stratified into two groups, the FGFR2/c-Jun/YAP1 co-activation group (28.94%) and the inactivation group (71.06%). The representative cases were demonstrated by different resolutions (Fig. 6d). By IHC analysis, some cases showed co-overexpression of FGFR2, c-Jun, and YAP1, while some cases were negative for these three proteins. The membrane/cytoplasmic FGFR2, nuclear c-Jun, and nuclear YAP1 were co-counted for survival analysis. The FGFR2–c-Jun–YAP1 co-activation group (79 out of 273) was positively correlated with poor disease specific. We further analyzed the differential power of the subgrouping in intestinal and diffuse subtypes, respectively. In whatever type of GC, the co-positive group is significantly associated with an unfavorable outcome compared with other cases (Fig. 6e). The stratification conveys a promising therapeutic target to the precision intervention of GC.

### Co-administration of small molecules for targeting the FGFR2–YAP1 axis

**Fig. 7:**
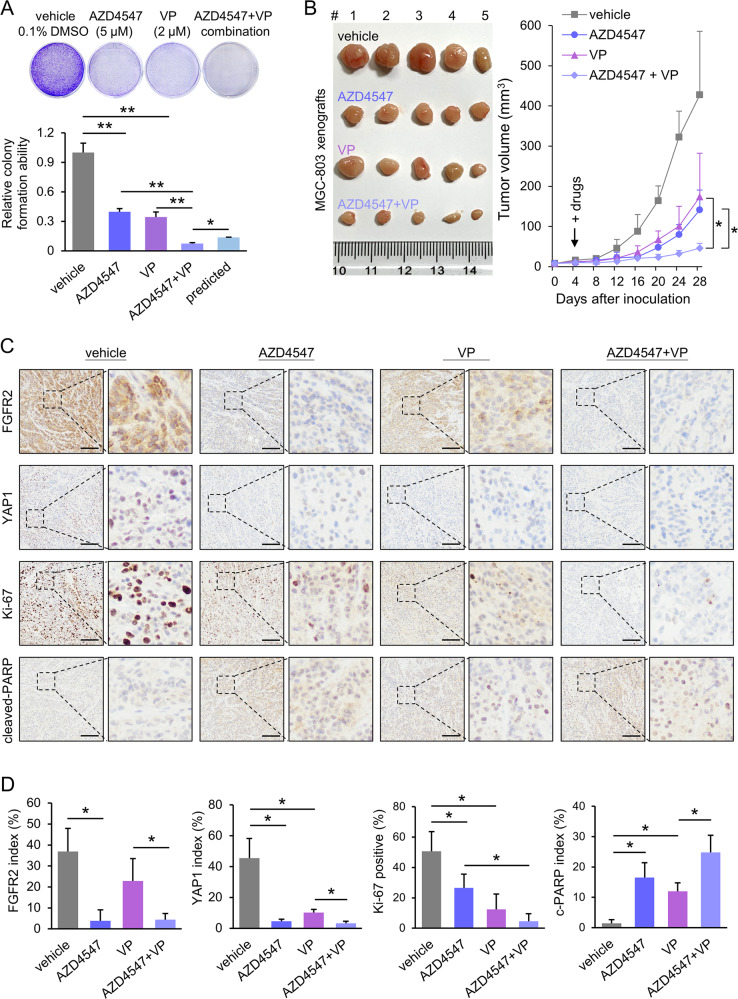
Targeting the FGFR2–c-Jun–YAP1 axis by small molecules. **a** Colony formation assays of MGC-803 administrated with FGFR2 inhibitor AZD4547 (5 µM) or YAP1 inhibitor verteporfin (VP, 2 µM) alone, or in combination (*p < 0.05; **p < 0.001). **b** Co-administration of VP and AZD4547 synergistically inhibited MGC-803-derived tumor growth compared with the effects of single AZD4547 or VP treatment (*p < 0.05). The xenograft sizes of each group were measured every 4 days. 0.1% DMSO in PBS as the vehicle control. **c** Representative IHC images of FGFR2, YAP1, Ki-67 in different MGC-803 xenograft sections (scale bar, 100 μm). **d** Expression levels of the indicated biomarkers were quantified by ImageJ (*n* = 3 areas; *p < 0.05).

To evaluate the effect of targeting the FGFR2–YAP1 axis, small molecules targeting FGFR2 and YAP1 were employed individually or in combination. Verteporfin (VP) is a small molecule blocking the YAP1–TEAD complex. The dose-response of VP was tested in MGC-803 (Supplementary Fig. S1F). In vitro combinational effect was tested by monolayer colony formation assay. The MGC-803 cells were treated with either AZD4547 (5 µM) or VP (2 µM) alone, or in combination (cells were treated with 5 µM AZD4547 for 24 h and then added with 2 µM VP for another 24 h). In total, 0.1% DMSO in PBS treatment was used as vehicle control. The predicted value of the combined group was calculated by multiplying the relative colony formation ability of the AZD4547 treated cells by that of VP treated cells. The effect was deemed synergistic when the observed value was less than the predicted value (Fig. 7a). In vivo synergistic effect was evaluated in xenograft formation models. Co-administration of MGC-803-derived xenografts with VP (a YAP1 inhibitor) and AZD4547 exerted a synergetic effect on tumor growth inhibition (Fig. 7b). IHC for FGFR2, YAP1, Ki-67, and cleaved PARP expression in these xenografts was performed and signals were captured under a microscope (Fig. 7c). The expression analysis manifested that the protein level of FGFR2, YAP1, and Ki-67 was further decreased by the combinational treatment of AZD4547 and VP, compared with the single treatment groups. While the expression of cleaved PARP was increased by the co-administration (*n* = 3, Fig. 7d). This demonstrates co-targeting FGFR2 and its downstream effector YAP1 might serve as a potential therapeutic strategy in GC.

**Fig. 8:**
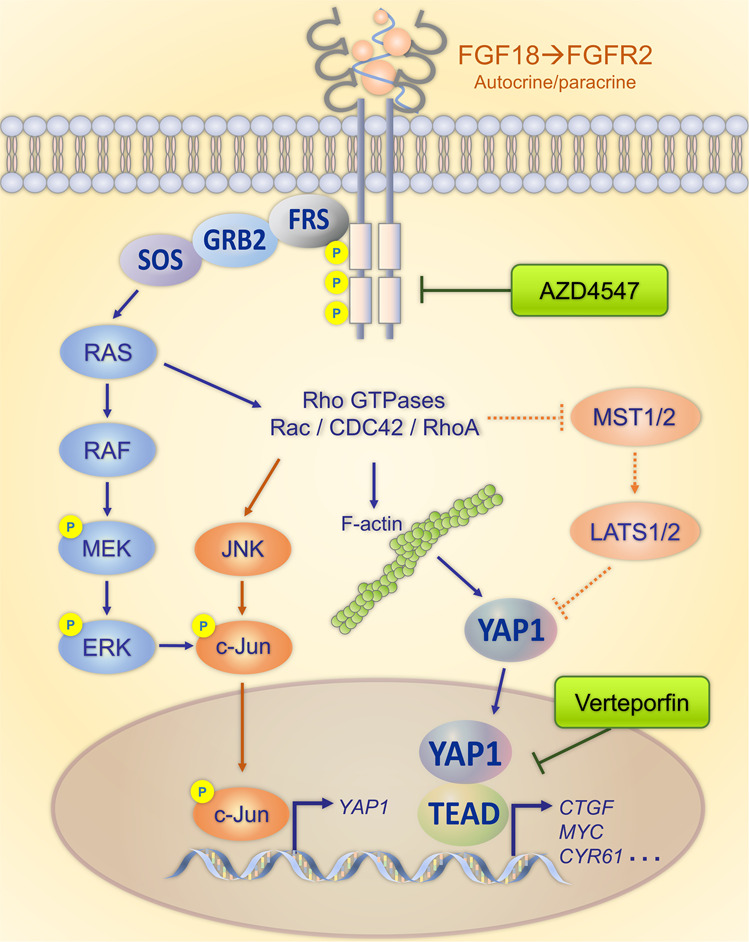
The FGF18–FGFR2 signaling activates YAP1 expression through MAPK–c-Jun cascade. FGF18–FGFR2 regulate the cytoskeleton and YAP1 activation, while Hippo signaling pathway is independent. As a transcription co-activator, YAP1 binds with TEADs to regulate the oncogenic downstream expression. Co-targeting FGFR2 and YAP1 might serves as a promising therapeutic strategy for the FGFR2–c-Jun–YAP1 co-activation GC cases.

In summary, we demonstrated the overexpression of FGFR2 in GC as well as its promoting role in gastric carcinogenesis. FGF18–FGFR2 axis promotes gastric progression by activating ERK–MAPK signaling. c-Jun is confirmed as a TF of YAP1. Serving as a potential downstream effector of FGF18–FGFR2, YAP1 transduces the oncogenic FGFR2 signaling into transcriptional events. F-actin cytoskeleton changes, which serves as a mechanotransduction mechanism for YAP1 nuclear translocation, is also regulated by FGF18–FGFR2. Co-activation of the FGFR2–c-Jun–YAP1 is identified in a group of GC cases and predicts unfavorable clinical outcome. A combination blockade of FGFR2 and YAP1 may benefit more GC patients undergoing targeted therapy (Fig. 8).

## Discussion

The oncogenic roles and clinical implications of FGF–FGFR family members have been dissected in various types of cancer. The FGF–FGFR signaling promotes cancer progression by the crosstalk with several canonical signaling pathways, contributing to the survival and metastasis of tumor cells [23, 28–30].

The therapeutic implication of FGFR2 in GC has been investigated for years. Co-occurrence of receptor tyrosine kinases and FGFR2 not only promotes GC progression but also leads to the acquisition of drug resistance. For instance, activation of epidermal growth factor receptor, human epidermal growth factor receptor 3, and MET induces drug resistance in GC cells with FGFR2 amplification [31]. On the other hand, signaling pathways such as ERK–MAPK [32], PI3K–Akt–mTOR [33], PLCγ–PKC [34], and JAK–STAT3 [35] are highlighted as canonical mechanisms involved in the oncogenic FGFR regulatory network. Although the copy number variation of FGFR2 is reported in GC, the detailed mechanism for its downstream signaling regulation is not unraveled. Identification of crucial downstream signaling of FGFR2 signaling in GC becomes imperative. In this study, we focused on deciphering molecular networks of FGFR2 and providing potential therapeutic strategies for GC. Among the four major FGFR members, FGFR2 shows the highest expression across multiple GC cell lines. The proportion of *FGFR2* amplification cases in the Hong Kong cohort is relatively higher in diffuse type GC than it is in intestinal type GC, concordant with other reports [21, 36, 37]. Apart from gene amplification, there is a proportion of GC patients harboring FGFR2 overexpression. Our results indicated that FGFR2 mRNA abundance significantly correlates with poor clinical outcomes. A previous meta-analysis also highlighted the pathological and prognostic importance of FGFR2 protein overexpression in GC patients of multiple sources [38]. Thus, from both mRNA level and protein level, FGFR2 overexpression is supposed to be a promising biomarker for the prognosis of GC. As to the functional role, depletion of FGFR2 leads to suppression of tumor growth by inducing G1 phase cycle arrest and apoptosis. We confirmed the ERK–MAPK as the main downstream signaling pathway of FGFR2. By RNA-seq analysis, cells with FGF18 stimulation or FGFR2 depletion revealed a subset of Hippo-YAP1 genes was dysregulated. YAP1, the key component of the Hippo-YAP1 pathway, was revealed as a potent downstream effector regulated by FGF18–FGFR2 signaling. Previously, the oncogenic role of YAP1 has been well characterized by us and others [39, 40]. The aberrantly overexpressed YAP1 accumulates in the nuclei and binds with TEAD TF to activate the expression of CTGF and c-Myc. In this study, we provide the first evidence that YAP1 is regulated by the c-Jun TF, which functions as the well-known downstream of FGF–FGFR–MAPK signaling in GC development [41]. The elucidation of novel mechanisms provides a potential for developing combinational therapeutic strategies.

To effectively targeting FGFR-related signaling, specific monoclonal antibodies and small-molecule inhibitors have been developed. The small molecules can be divided into non-selective and selective inhibitors. Among all the selective FGFR inhibitors, AZD4547 shows potent preclinical activity in gastric adenocarcinomas with *FGFR2* amplification and other gastrointestinal tumors [42, 43]. However, its efficacy has been questioned by several studies. In an open-label randomized phase II trial, the efficacy of AZD4547 versus paclitaxel as a second-line treatment was compared in patients with advanced gastric adenocarcinoma. *FGFR2* polysomy or gene amplification was examined by fluorescence in situ hybridization in patient samples before drug treatment. Unexpectedly, progression-free survival was not significantly increased by AZD4547 [24, 31, 36]. Heterogeneity within the tumors is an important factor. Besides, alternative molecular mechanisms might be involved in the acquisition of drug resistance as well. Thus, elucidation of detailed and comprehensive signaling crosstalks and transduction of FGF–FGFR may develop novel therapeutic strategies. Co-activation of the FGFR2–c-Jun–YAP1 signaling cascade was identified in a subgroup of GC patients with worse survival. Co-targeting YAP1 and FGFR2 by VP and AZD4547 significantly improved the antineoplastic efficacies in animal models. Hopefully, the proposed combinational targeting may convey a novel therapeutic strategy for GC patients in this personalized medicine era.

## Materials and methods

### GC cell lines and primary samples

The source of human GC cell lines and tissue microarray have been described previously [44]. Human sample usage was approved by the Joint Chinese University of Hong Kong—New Territories East Cluster Clinical Research Ethics Committee (CREC Ref. No.: 2019.406).

### Chromogenic in situ hybridization (CISH)

FGFR2 probe (ZytoDot 2C SPEC FGFR2/CEN 10 Probe, C-3056; ZytoDot 2C CISH Implementation Kit, C-3044, Zytovision, Germany) was used to check the copy number gain and gene amplification in primary GC samples (Hong Kong cohort).

### Quantitative real-time polymerase chain reaction (qRT-PCR)

Total RNA extraction and qRT-PCR have been described [44]. The primer sequences used in this study were listed in Supplementary Table S5.

### Western blot analysis

Assays were performed as a previous study [44]. Antibodies were from Cell Signaling (Danvers, MA), including Phospho-FGFR (Tyr653/654) (#3471), Phospho-MEK1/2 (#9121), Phospho-p44/42 MAPK (#9106), p44/42 MAPK (#4695), Cyclin D1 (#2978), p21 (#2946), p27 (#2552), Phospho-Rb (Ser807/811) (#9308), cleaved PARP (Asp214) (#9541), PARP (#9542), LATS1 (#9153), LATS2 (#5888) Phospho c-Jun (Ser73) (#3270), c-Jun (#9165), c-Myc (#9402); Abcam (MA, US), including anti-FGFR2 (ab58201), and anti-YAP1 (ab52771); Immunoway (TX, US), including Bcl2 (YM3041) and β-Actin (YM3115); Santa Cruz (TX, US) including CTGF (sc-14939) and TEAD4 (TEF-3, sc-101184). The other antibodies include Phospho-LATS1/2 (Ser909/872) (AP0904, Abclonal), N-cadherin (#33-3900, ZYMED).

### Immunohistochemistry (IHC) and immunofluorescence (IF)

IHC was conducted on the TMA and xenograft samples using Ventana NexES automated Stainer (Ventana Corporation). Primary antibodies for IF include YAP1 and α-Tubulin. Alexa Fluor 488 Phalloidin (Invitrogen) used as a F-actin indicator. Nuclei were counterstained with DAPI (Sigma-Aldrich). Images were acquired by confocal microscope Carl Zeiss LSM880 (Gottingen, Germany).

### In vitro functional studies

GC cells were transfected with either QIAGEN FlexiTube siFGFR2 (SI02623047 and SI04948902), siJUN (SI00300580), or siScramble control (SI03650318). Detailed functional test procedures were previously described [44]. Recombinant human FGF18 (rhFGF18) (100-28, PeproTech) was employed to induce activation of FGFR.

### RNA sequencing analysis

MGC-803 was used for screening the expression-altered genes after rhFGF18 stimulation or FGFR2 depletion. RNA-seq was performed by HiSeq-PE150 (Medikonia). Bioinformatics analysis revealed the alteration of biological processes by GO enrichment analysis [45]. Primer sequences for the validation of candidate genes were listed in Supplementary Table S5.

### Immunoprecipitation assays

For chromatin immunoprecipitation (ChIP), the promoter region of YAP1 was defined within 2000 base pairs upstream of the transcription start site and the sequence was obtained from the UCSC Table Browser [46]. Based on TRANSFAC (version 8.3), two putative binding sites of c-Jun were predicted at the region [47, 48]. Crosslinking, lysis, and chromatin shearing were performed as previously described [25]. Targeted fragments (300–500 base pairs) were pulled down by Magnetic Dynabeads Protein G (1004, Life Tech), with c-Jun antibody or Normal IgG antibody. Primers for binding site sequences are listed in Supplementary Table S5. For co-immunoprecipitation, 500 µg cell lysate was used for immunoprecipitation with 20 µl magnetic beads, bonding with YAP1 antibody or normal IgG antibodyA light chain-specific secondary antibody (ab99632, Abcam) were used to avoid the signal of the heavy chain (~50 kDa).

### Drug sensitivity test

Cells were seeded (5000 cells/well). Varying doses of 5-Fluorouracil (5-FU: 0, 10, 20, 50, 100, 250 μg/ml) or AZD4547 (0, 0.1, 1, 2, 5, 10 μM) were added. After 48 h, cell viability was evaluated by using Cell Counting Kit, CCK-8 (Dojindo, Kumamoto, Japan). Data were recorded at 450 nm absorbance and proportioned to the number of living cells.

### In vivo xenograft formation assay

MGC-803-derived xenografts was used for the efficacy appraisal of combinational administration. Total 20 mice were subcutaneously injected with 10^7^ MGC-803 cells for each mouse. After 4 days’ inoculation, when the xenografts were palpable (~20 mm^3^), the mice were randomly divided as four groups (*n* = 5/group) and then treated with vehicle, AZD4547 (5 mg/kg/day by oral gavage), VP (20 mg/kg by intraperitoneal injection every other day), or the combination of AZD4547 and VP, respectively, for consecutive 24 days. Tumors size were measured every 4 days by digital calipers. The mice were sacrificed 28 days after the inoculation for immunostaining analysis. All the animal handling was approved by the Animal Experimentation Ethics Committee, The Chinese University of Hong Kong (Ref. No. 19-042-GRF).

### Statistical analysis

All statistical analysis was performed using Graphpad Prism (version 8.0.1) and SPSS software (version 22.0; SPSS Inc). The student’s *t* test was used to compare the expression level of FGFR2 in the TCGA cohort, as well as the functional differences between siRNA-transfectants and controls. Cox regression was applied to indicate the survival rate for each parameter. For those variables that were found statistically significant in the univariate survival analysis, the Cox proportional hazards model with the likelihood ratio statistics was employed to assess them for multivariate survival analysis. The half-maximal inhibitory concentration (IC_50_) was calculated under a nonlinear regression model and statistical significance among treatment groups was determined by ANOVA. Two-tailed *p* values of <0.05 were deemed as statistically significant and those of <0.001 were highly statistically significant.

## Supplementary information

Supplementary Figure S1

Supplementary Table S1

Supplementary Table S2

Supplementary Table S3

Supplementary Table S4

Supplementary Table S5
