# Supplementary figures and images for: FGF18–FGFR2 signaling triggers the activation of c-Jun–YAP1 axis to promote carcinogenesis in a subgroup of gastric cancer patients and indicates translational potential
Source: Oncogene. 2020 Sep 15;39(43):6647–63. doi: 10.1038/s41388-020-01458-x (PMC7581496; doi:10.1038/s41388-020-01458-x)

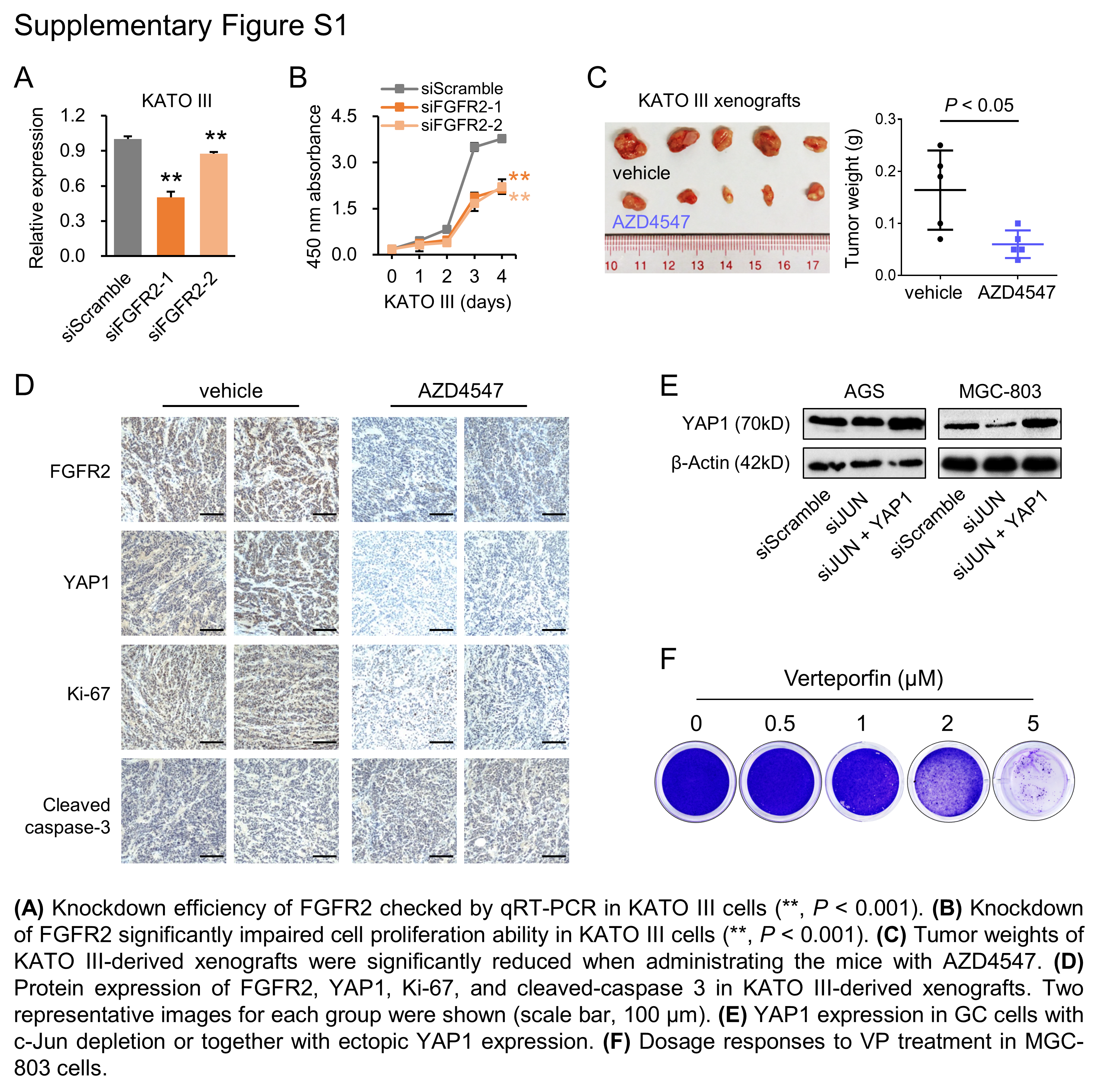

Supplement: Supplementary file 1 — Supplementary Figure S1 [file 41388_2020_1458_MOESM1_ESM.tif]
