# Supplementary material for: FGF18–FGFR2 signaling triggers the activation of c-Jun–YAP1 axis to promote carcinogenesis in a subgroup of gastric cancer patients and indicates translational potential
Source: Oncogene. 2020 Sep 15;39(43):6647–63. doi: 10.1038/s41388-020-01458-x (PMC7581496; doi:10.1038/s41388-020-01458-x)
Supplement: Supplementary file 2 — Supplementary Table S1 [file 41388_2020_1458_MOESM2_ESM.docx]

**Supplementary Table S1.** Correlation of FGFR2 expression in GC with other clinicopathologic features (significant *P*-value in bold and Italic format). The case number and percentage counted were shown in the table.

|  |  | Gastric cancer (n = 265) | |  |
| --- | --- | --- | --- | --- |
|  |  | Expression < 5% | Expression > 5% | *P*-value |
| Sex | M | 70 (40.5%) | 103 (59.5%) | 0.278 |
|  | F | 43 (47.3%) | 48 (52.7%) |  |
| Age | <=60 | 49 (51%) | 47 (49%) | 0.051 |
|  | >60 | 64 (38.1%) | 104 (61.9%) |  |
| Type | Intestinal | 58 (43.1%) | 83 (58.9%) | 0.651 |
|  | Diffuse | 54 (44.3%) | 68 (55.7%) |  |
| Grade | 1 | 3 (33.3%) | 6 (66.7%) | 0.492 |
|  | 2 | 37 (38.9%) | 58 (61.1%) |  |
|  | 3 | 72 (45.3%) | 87 (54.7%) |  |
| Stage | 1 | 31 (53.4%) | 27 (46.6%) | 0.133 |
|  | 2 | 16 (50%) | 16 (50%) |  |
|  | 3 | 33 (38.8%) | 52 (61.2%) |  |
|  | 4 | 32 (36.4%) | 56 (63.6%) |  |
| Stage (T) | 1 | 20 (54.1%) | 17 (45.9%) | 0.056 |
|  | 2 | 36 (49.3%) | 37 (50.7%) |  |
|  | 3 | 49 (35.0%) | 91 (65.0%) |  |
|  | 4 | 7 (53.8%) | 6 (46.2%) |  |
| Stage (N) | 0 | 29 (49.2%) | 30 (50.8%) | ***0.029*** |
|  | 1 | 38 (53.5%) | 33 (46.5%) |  |
|  | 2 | 26 (34.2%) | 50 (65.8%) |  |
|  | 3 | 19 (33.3%) | 38 (66.7%) |  |
| Stage (M) | 0 | 97 (43.5%) | 126 (56.5%) | 0.412 |
|  | 1 | 15 (37.5%) | 25 (62.5%) |  |
| Lymph Node | 0 | 29 (49.2%) | 30 (50.8%) | 0.237 |
|  | 1 | 83 (40.7%) | 121 (59.3%) |  |
| *H. pylori* | Absence | 50 (41.3%) | 71 (58.7%) | 0.987 |
|  | Presence | 54 (41.5%) | 76 (58.5%) |  |
