# Supplementary material for: FGF18–FGFR2 signaling triggers the activation of c-Jun–YAP1 axis to promote carcinogenesis in a subgroup of gastric cancer patients and indicates translational potential
Source: Oncogene. 2020 Sep 15;39(43):6647–63. doi: 10.1038/s41388-020-01458-x (PMC7581496; doi:10.1038/s41388-020-01458-x)
Supplement: Supplementary file 3 — Supplementary Table S2 [file 41388_2020_1458_MOESM3_ESM.docx]

**Supplementary Table S2.** Univariate and multivariate Cox regression analysis of the association between clinicopathologic characteristics and disease specific survival in GC patients (n = 265, significant *P*-value in bold and Italic format).

|  | Univariate analysis | Multivariate analysis |
| --- | --- | --- |
| Sex | 0.285 |  |
| Age | *0.039* | *< 0.001* |
| Type | *< 0.001* | 0.645 |
| Grade | *0.006* | 0.748 |
| Stage | *< 0.001* |  |
| Stage (T) | *< 0.001* | *< 0.001* |
| Stage (N) | *< 0.001* | *< 0.001* |
| Stage (M) | *< 0.001* | *< 0.001* |
| Lymph Node | *< 0.001* |  |
| *H. pylori* | 0.21 |  |
| FGFR2 | *0.025* | 0.612 |
