# Supplementary material for: FGF18–FGFR2 signaling triggers the activation of c-Jun–YAP1 axis to promote carcinogenesis in a subgroup of gastric cancer patients and indicates translational potential
Source: Oncogene. 2020 Sep 15;39(43):6647–63. doi: 10.1038/s41388-020-01458-x (PMC7581496; doi:10.1038/s41388-020-01458-x)
Supplement: Supplementary file 4 — Supplementary Table S3 [file 41388_2020_1458_MOESM4_ESM.pdf]

Term	Description	log10 p value
	After FGF18 stimulation	
GO:1904035	Apoptotic process	-2.03042087
GO:0007265	Ras protein signal transduction	-2.14504325
GO:0051893	Focal adhesion assembly	-2.37716051
GO:0050673	Epithelial cell proliferation	-2.39586478
GO:0043405	MAP kinase activity	-2.82573139
GO:0050679	Epithelial cell proliferation	-2.88900007
GO:0034764	Transmembrane transport	-3.05962753
GO:0043087	GTPase activity	-3.14663392
GO:0001933	Protein phosphorylation	-3.72073299
GO:0071901	Serine/threonine kinase activity	-3.85761015
	After FGFR2 depletion	
GO:0072331	signal transduction by p53 class mediator	-4.92324815
GO:0008630	intrinsic apoptotic signaling pathway in response to DNA damage	-4.49499288
GO:0000188	inactivation of MAPK activity	-3.62629707
GO:0010801	negative regulation of peptidyl-threonine phosphorylation	-3.16917026
GO:0010469	regulation of signaling receptor activity	-3.08702871
GO:0040036	regulation of fibroblast growth factor receptor signaling pathway	-3.02557716
GO:0060572	morphogenesis of an epithelial bud	-3.00230226
GO:0030330	DNA damage response, signal transduction by p53 class mediator	-2.86082711
GO:0006469	negative regulation of protein kinase activity	-2.74779779
GO:0008543	fibroblast growth factor receptor signaling pathway	-2.61514982

Name	Negative c	FGF18 stin	Fold_chan
CTGF	6.44607	25.8655	4.0126
CCND1	35.1298	82.4122	2.345934
AXIN2	0.4516	0.936739	2.074267
BMPR1A	4.69175	8.94571	1.906689
FZD9	0.838035	1.51857	1.81206
WNT10B	6.16492	10.0612	1.632008
TGFB3	4.22929	6.22395	1.47163
CCND3	81.1767	117.203	1.443801
WWTR1	28.5973	40.4764	1.415392
MYC	227.661	320.201	1.406482
TGFBR2	8.53484	11.6396	1.363775
CSNK1E	56.9823	76.6406	1.34499
AXIN1	26.7104	35.3312	1.322751
TGFB1	78.0128	102.444	1.313169
BMP6	32.8906	42.0978	1.279934
PPP1CB	63.9148	81.6755	1.277881
CTNNB1	73.1472	93.0466	1.272046
TGFBR1	6.95864	8.6304	1.240242
SMAD2	59.275	72.7059	1.226586
YAP1	29.029	35.273	1.215095
TEAD1	30.884	36.2101	1.172455
TEAD4	63.0498	73.7792	1.170173
GSK3B	20.1235	19.2484	0.956514
MOB1A	31.8985	30.0048	0.940634
LIMD1	6.93267	5.22707	0.753976
BMPR2	2.1516	1.42069	0.660295
TP73	1.80726	0.576355	0.318911
PPP2R2B	0.886808	0.217429	0.245182
AMOT	1.8311	0.256726	0.140203

Name	Negative c	FGFR2 dep	Fold_chang
GLI2	15.53	0.00322	0.05
MYC	110.42	38.6664	0.350176
BIRC5	92.5024	37.7617	0.408224
TEAD4	73.8106	30.2194	0.409418
NF2	18.3365	9.09155	0.495817
CCND3	86.2583	44.2206	0.512653
ACTG1	1911.87	1095.14	0.572811
AXIN1	82.2195	48.9223	0.595021
RASSF1	30.1492	19.0013	0.630242
AREG	24.4879	16.3135	0.666186
TGFB1	3.26799	2.23866	0.685027
TEAD3	21.4463	15.6654	0.730448
SOX2	40.247	29.5577	0.734408
MOB1A	56.9514	43.5733	0.765096
TEAD1	10.0909	8.08037	0.800758
YAP1	78.1803	64.3692	0.823343
TGFBR1	6.64364	5.8178	0.875695
GSK3B	15.7669	16.3312	1.03579
CTGF	0.380858	0.410524	1.077893
LATS1	0.628989	0.689521	1.096237
AJUBA	30.4477	34.882	1.145637
STK3	5.21984	6.10999	1.170532
LATS2	7.25073	8.98466	1.239139
CDH1	44.1497	56.5375	1.280586
TEAD2	21.916	28.5526	1.30282
BIRC2	17.6692	26.7233	1.512423
AMOT	3.81218	5.93664	1.557282
RASSF6	3.3801	5.78161	1.710485
AXIN2	56.6644	107.251	1.89274
BTRC	3.58188	9.9947	2.79035
SAV1	0.094093	0.301371	3.202912
FRMD1	0.036793	0.258601	7.028538
TP73	0.056055	0.492407	8.784323
ITGB2	0.19172	2.77736	14.48654
PPP2R2B	0.00053	0.057174	107.7948
