# Supplementary material for: FGF18–FGFR2 signaling triggers the activation of c-Jun–YAP1 axis to promote carcinogenesis in a subgroup of gastric cancer patients and indicates translational potential
Source: Oncogene. 2020 Sep 15;39(43):6647–63. doi: 10.1038/s41388-020-01458-x (PMC7581496; doi:10.1038/s41388-020-01458-x)
Supplement: Supplementary file 5 — Supplementary Table S4 [file 41388_2020_1458_MOESM5_ESM.pdf]

Predicted transcription factors binding on YAP1 promoter region by JASPAR database

Matrix ID	Name	Score	Relative score	Sequence ID	Start	End	Strand	Predicted sequence
MA0149.1	EWSR1-FLI1	24.48	0.905	hg38_knownG	1763	1780	-	gaaagggaggaaggaagg
MA0149.1	EWSR1-FLI1	19.50	0.853	hg38_knownG	800	817	+	ggaagaaagaaaggaaga
MA0149.1	EWSR1-FLI1	19.20	0.850	hg38_knownG	1759	1776	-	gggaggaaggaaggaaca
MA1653.1	ZNF148	18.93	1.000	hg38_knownG	637	648	+	cccccctccccc
MA0149.1	EWSR1-FLI1	18.74	0.845	hg38_knownG	747	764	-	ggtggggaggaaggaagg
MA1653.1	ZNF148	17.70	0.979	hg38_knownG	417	428	-	ttcccctccccc
MA1584.1	ZIC5	17.32	0.930	hg38_knownG	1256	1271	-	gcaccccccgccgggc
MA1522.1	MAZ	16.82	1.000	hg38_knownG	637	647	+	cccccctcccc
MA1522.1	MAZ	16.82	1.000	hg38_knownG	989	999	+	cccccctcccc
MA1653.1	ZNF148	16.36	0.957	hg38_knownG	989	1000	+	cccccctcccct
MA0137.3	STAT1	16.29	0.983	hg38_knownG	333	343	+	cttccaggaaa
MA1513.1	KLF15	16.27	0.977	hg38_knownG	1571	1581	+	cccccgccccg
MA0697.1	ZIC3	16.21	0.906	hg38_knownG	1256	1270	-	caccccccgccgggc
MA0149.1	EWSR1-FLI1	16.16	0.818	hg38_knownG	431	448	+	gggaggggggaaggaaag
MA0732.1	EGR3	15.90	0.922	hg38_knownG	1586	1600	+	ctccgcccccgccct
MA0751.1	ZIC4	15.60	0.927	hg38_knownG	1256	1270	-	caccccccgccgggc
MA1522.1	MAZ	15.41	0.979	hg38_knownG	430	440	-	cccccctcccg
MA1522.1	MAZ	15.34	0.978	hg38_knownG	418	428	-	ttcccctcccc
MA0162.4	EGR1	15.27	0.927	hg38_knownG	1586	1599	+	ctccgcccccgccc
MA0698.1	ZBTB18	15.26	0.948	hg38_knownG	176	188	+	cgtacagatgtgg
MA0508.3	PRDM1	15.02	0.984	hg38_knownG	1735	1745	+	ccctttctctt
MA0528.2	ZNF263	14.99	0.963	hg38_knownG	752	763	-	gtggggaggaag
MA0039.4	KLF4	14.87	0.975	hg38_knownG	737	748	+	ttccccaccccc
MA0039.4	KLF4	14.83	0.974	hg38_knownG	756	767	+	ctccccacccct
MA0733.1	EGR4	14.69	0.894	hg38_knownG	1586	1601	+	ctccgcccccgccctg
MA1522.1	MAZ	14.68	0.968	hg38_knownG	412	422	-	tccccctcccg
MA0131.2	HINFP	14.66	0.951	hg38_knownG	1234	1245	-	gcacgtccgcgg
MA0149.1	EWSR1-FLI1	14.63	0.802	hg38_knownG	435	452	+	ggggggaaggaaagaaag
MA0149.1	EWSR1-FLI1	14.63	0.802	hg38_knownG	1767	1784	-	aaaagaaagggaggaagg
MA1511.1	KLF10	14.62	0.959	hg38_knownG	610	620	+	aacacacccac
MA1107.2	KLF9	14.62	0.889	hg38_knownG	608	623	+	gcaacacacccacaca
MA1653.1	ZNF148	14.47	0.924	hg38_knownG	429	440	-	cccccctcccgt
MA0144.2	STAT3	14.43	0.972	hg38_knownG	333	343	-	tttcctggaag
MA1522.1	MAZ	14.38	0.964	hg38_knownG	1635	1645	-	agcccctcccg
MA0836.2	CEBPD	14.36	0.944	hg38_knownG	701	713	-	gcttgcacaatgc
MA0144.2	STAT3	14.35	0.971	hg38_knownG	333	343	+	cttccaggaaa
MA1513.1	KLF15	14.32	0.949	hg38_knownG	1591	1601	+	cccccgccctg
MA0039.4	KLF4	14.31	0.963	hg38_knownG	961	972	+	acccccacccca
MA0162.4	EGR1	14.29	0.910	hg38_knownG	611	624	+	acacacccacacac
MA1564.1	SP9	14.22	0.945	hg38_knownG	827	838	+	agcacgcccccc
MA1653.1	ZNF148	14.19	0.920	hg38_knownG	761	772	+	cacccctcctcc
MA0696.1	ZIC1	14.15	0.906	hg38_knownG	1257	1270	-	caccccccgccggg
MA0812.1	TFAP2B(var.2)	14.03	0.963	hg38_knownG	1269	1279	-	cgccgcaggca
MA1596.1	ZNF460	13.97	0.851	hg38_knownG	1411	1426	+	gcttctgcctcccccg
MA0599.1	KLF5	13.89	0.980	hg38_knownG	738	747	+	tccccacccc
MA0599.1	KLF5	13.89	0.980	hg38_knownG	757	766	+	tccccacccc
MA0835.2	BATF3	13.84	0.953	hg38_knownG	96	106	-	gttgactcata
MA1653.1	ZNF148	13.84	0.914	hg38_knownG	411	422	-	tccccctcccgt
MA1135.1	FOSB::JUNB	13.76	0.963	hg38_knownG	35	44	-	gatgactaat
MA1650.1	ZBTB14	13.74	0.933	hg38_knownG	1425	1436	+	cgacgcgcaccc
MA1522.1	MAZ	13.63	0.953	hg38_knownG	761	771	+	cacccctcctc
MA1653.1	ZNF148	13.63	0.910	hg38_knownG	1067	1078	-	tgcccctctccc
MA0462.2	BATF::JUN	13.62	0.955	hg38_knownG	96	106	-	gttgactcata
MA1141.1	FOS::JUND	13.61	0.936	hg38_knownG	33	45	+	gaattagtcatcg
MA1138.1	FOSL2::JUNB	13.60	0.959	hg38_knownG	35	44	-	gatgactaat
MA0599.1	KLF5	13.58	0.976	hg38_knownG	1571	1580	+	cccccgcccc

MA1128.1	FOSL1::JUN	13.56	0.944	hg38_knownG	33	45	-	cgatgactaattc
MA0155.1	INSM1	13.55	0.928	hg38_knownG	1432	1443	-	ggtcagggggtg
MA0506.1	NRF1	13.54	0.944	hg38_knownG	1623	1633	+	gcggctgcgcc
MA0599.1	KLF5	13.54	0.976	hg38_knownG	962	971	+	cccccacccc
MA1634.1	BATF	13.54	0.956	hg38_knownG	96	106	-	gttgactcata
MA1144.1	FOSL2::JUND	13.49	0.954	hg38_knownG	35	44	-	gatgactaat
MA0137.3	STAT1	13.47	0.944	hg38_knownG	333	343	-	tttcctggaag
MA0516.2	SP2	13.43	0.856	hg38_knownG	734	750	+	cttttccccaccccctt
MA1548.1	PLAGL2	13.41	0.966	hg38_knownG	567	576	-	tgggcccctt
MA0814.2	TFAP2C(var.2)	13.40	0.920	hg38_knownG	1268	1281	+	gtgcctgcggcgcc
MA1102.2	CTCFL	13.37	0.927	hg38_knownG	1277	1288	-	cccaggcggcgc
MA0099.3	FOS::JUN	13.36	0.957	hg38_knownG	35	44	+	attagtcatc
MA0599.1	KLF5	13.31	0.973	hg38_knownG	418	427	-	tcccctcccc
MA0613.1	FOXG1	13.31	0.986	hg38_knownG	325	332	+	ataaacaa
MA1512.1	KLF11	13.29	0.938	hg38_knownG	610	620	+	aacacacccac
MA1137.1	FOSL1::JUNB	13.27	0.953	hg38_knownG	33	45	-	cgatgactaattc
MA0102.4	CEBPA	13.26	0.907	hg38_knownG	701	714	-	ggcttgcacaatgc
MA1653.1	ZNF148	13.25	0.904	hg38_knownG	1570	1581	+	tcccccgccccg
MA0471.2	E2F6	13.25	0.926	hg38_knownG	816	828	+	gaaggagggaaag
MA1541.1	NR6A1	13.22	0.858	hg38_knownG	1356	1372	-	cccagggccaagggcag
MA0599.1	KLF5	13.13	0.970	hg38_knownG	1591	1600	+	cccccgccct
MA1130.1	FOSL2::JUN	13.12	0.945	hg38_knownG	33	44	+	gaattagtcatc
MA0528.2	ZNF263	13.05	0.924	hg38_knownG	1768	1779	-	aaagggaggaag
MA1569.1	TFAP2E	13.01	0.975	hg38_knownG	1269	1279	+	tgcctgcggcg
MA0599.1	KLF5	12.96	0.968	hg38_knownG	638	647	+	ccccctcccc
MA0599.1	KLF5	12.96	0.968	hg38_knownG	990	999	+	ccccctcccc
MA0489.1	JUN(var.2)	12.90	0.931	hg38_knownG	35	48	-	aagcgatgactaat
MA0842.2	NRL	12.88	0.919	hg38_knownG	401	413	+	ataaatgcttacg
MA0746.2	SP3	12.86	0.915	hg38_knownG	826	838	+	aagcacgcccccc
MA0741.1	KLF16	12.84	0.921	hg38_knownG	638	648	+	ccccctccccc
MA1569.1	TFAP2E	12.83	0.973	hg38_knownG	1269	1279	-	cgccgcaggca
MA1116.1	RBPJ	12.76	0.990	hg38_knownG	648	657	-	gctgggaaag
MA1134.1	FOS::JUNB	12.75	0.931	hg38_knownG	34	45	+	aattagtcatcg
MA0517.1	STAT1::STAT2	12.70	0.867	hg38_knownG	1773	1787	+	tccctttcttttccc
MA1132.1	JUN::JUNB	12.68	0.953	hg38_knownG	35	44	-	gatgactaat
MA0769.2	TCF7	12.67	0.958	hg38_knownG	117	127	-	gcctttgataa
MA0872.1	TFAP2A(var.3)	12.65	0.904	hg38_knownG	1255	1267	+	tgcccggcggggg
MA1512.1	KLF11	12.64	0.928	hg38_knownG	827	837	+	agcacgccccc
MA0736.1	GLIS2	12.64	0.880	hg38_knownG	1257	1270	-	caccccccgccggg
MA0815.1	TFAP2C(var.3)	12.62	0.911	hg38_knownG	832	844	+	gccccccggggct
MA0872.1	TFAP2A(var.3)	12.61	0.904	hg38_knownG	832	844	-	agccccggggggc
MA0528.2	ZNF263	12.58	0.915	hg38_knownG	895	906	+	tctgggaggagt
MA0038.2	GFI1	12.57	0.924	hg38_knownG	877	888	+	caaatctcagcc
MA0872.1	TFAP2A(var.3)	12.52	0.902	hg38_knownG	832	844	+	gccccccggggct
MA0741.1	KLF16	12.51	0.915	hg38_knownG	610	620	+	aacacacccac
MA0490.2	JUNB	12.46	0.919	hg38_knownG	33	45	-	cgatgactaattc
MA0815.1	TFAP2C(var.3)	12.45	0.909	hg38_knownG	832	844	-	agccccggggggc
MA1513.1	KLF15	12.38	0.921	hg38_knownG	1046	1056	+	ggcccgcccga
MA0163.1	PLAG1	12.38	0.835	hg38_knownG	1022	1035	-	ggggcgctcgggag
MA1585.1	ZKSCAN1	12.34	0.930	hg38_knownG	250	259	-	ttagtaggtc
MA1646.1	OSR2	12.34	0.935	hg38_knownG	1409	1420	-	aggcagaagcca
MA1517.1	KLF6	12.31	0.943	hg38_knownG	609	619	+	caacacaccca
MA0471.2	E2F6	12.27	0.908	hg38_knownG	1723	1735	-	ggaggcggggaaa
MA0740.1	KLF14	12.27	0.884	hg38_knownG	826	839	+	aagcacgcccccg
MA0080.5	SPI1	12.27	0.819	hg38_knownG	1741	1760	-	caagaaaaggaaataaagag
MA0865.1	E2F8	12.25	0.841	hg38_knownG	1680	1691	+	tttccctccaac
MA1516.1	KLF3	12.24	0.927	hg38_knownG	1154	1164	-	gaccgtgccca
MA0477.2	FOSL1	12.24	0.924	hg38_knownG	33	45	-	cgatgactaattc

MA0477.2	FOSL1	12.23	0.924	hg38_knownG	95	107	-	ggttgactcatag
MA1106.1	HIF1A	12.23	0.980	hg38_knownG	1238	1247	+	ggacgtgcac
MA0741.1	KLF16	12.22	0.911	hg38_knownG	827	837	+	agcacgccccc
MA0491.2	JUND	12.21	0.918	hg38_knownG	95	107	-	ggttgactcatag
MA1548.1	PLAGL2	12.20	0.942	hg38_knownG	634	643	+	aggcccccct
MA0810.1	TFAP2A(var.2)	12.18	0.942	hg38_knownG	833	844	+	ccccccggggct
MA0812.1	TFAP2B(var.2)	12.17	0.935	hg38_knownG	1269	1279	+	tgcctgcggcg
MA0764.2	ETV4	12.15	0.957	hg38_knownG	750	759	-	ggaggaagga
MA0764.2	ETV4	12.15	0.957	hg38_knownG	1766	1775	-	ggaggaagga
MA0466.2	CEBPB	12.14	0.949	hg38_knownG	703	712	-	cttgcacaat
MA0815.1	TFAP2C(var.3)	12.13	0.905	hg38_knownG	1255	1267	+	tgcccggcggggg
MA0815.1	TFAP2C(var.3)	12.13	0.905	hg38_knownG	1255	1267	-	cccccgccgggca
MA0835.2	BATF3	12.12	0.921	hg38_knownG	34	44	-	gatgactaatt
MA0163.1	PLAG1	12.12	0.830	hg38_knownG	1594	1607	-	ggggcgcagggcgg
MA1146.1	NR1H4::RXRA	12.12	0.854	hg38_knownG	309	323	-	gagttctttcacccc
MA0491.2	JUND	12.11	0.916	hg38_knownG	33	45	-	cgatgactaattc
MA0073.1	RREB1	12.10	0.807	hg38_knownG	758	777	+	ccccacccctcctccactcc
MA0837.1	CEBPE	12.08	0.946	hg38_knownG	703	712	-	cttgcacaat
MA1515.1	KLF2	12.08	0.945	hg38_knownG	1590	1600	+	gcccccgccct
MA0074.1	RXRA::VDR	12.03	0.829	hg38_knownG	1430	1444	-	gggtcagggggtgcg
MA0039.4	KLF4	12.03	0.917	hg38_knownG	637	648	+	cccccctccccc
MA0741.1	KLF16	12.02	0.907	hg38_knownG	738	748	+	tccccaccccc
MA0490.2	JUNB	12.01	0.911	hg38_knownG	95	107	-	ggttgactcatag
MA0872.1	TFAP2A(var.3)	12.00	0.894	hg38_knownG	1364	1376	+	ggccctgggggggc
MA0462.2	BATF::JUN	11.98	0.923	hg38_knownG	34	44	-	gatgactaatt
MA1653.1	ZNF148	11.97	0.882	hg38_knownG	1727	1738	+	cccgcctcccct
MA1653.1	ZNF148	11.96	0.882	hg38_knownG	1634	1645	-	agcccctcccga
MA0039.4	KLF4	11.96	0.915	hg38_knownG	1590	1601	+	gcccccgccctg
MA0479.1	FOXH1	11.96	0.941	hg38_knownG	488	498	+	tctaatcaaca
MA0039.4	KLF4	11.93	0.915	hg38_knownG	1570	1581	+	tcccccgccccg
MA0033.2	FOXL1	11.92	0.994	hg38_knownG	325	331	+	ataaaca
MA1123.2	TWIST1	11.91	0.920	hg38_knownG	176	188	+	cgtacagatgtgg
MA0079.4	SP1	11.90	0.902	hg38_knownG	824	838	+	gaaagcacgcccccc
MA0810.1	TFAP2A(var.2)	11.90	0.938	hg38_knownG	1364	1375	-	ccccccagggcc
MA1142.1	FOSL1::JUND	11.89	0.967	hg38_knownG	35	44	-	gatgactaat
MA1128.1	FOSL1::JUN	11.88	0.913	hg38_knownG	95	107	-	ggttgactcatag
MA1141.1	FOS::JUND	11.88	0.906	hg38_knownG	95	107	+	ctatgagtcaacc
MA0764.2	ETV4	11.86	0.952	hg38_knownG	746	755	-	gaaggaaggg
MA1634.1	BATF	11.85	0.922	hg38_knownG	34	44	-	gatgactaatt
MA0495.3	MAFF	11.85	0.851	hg38_knownG	399	414	-	ccgtaagcatttattt
MA1105.2	GRHL2	11.85	0.896	hg38_knownG	363	374	-	aatacaggtttc
MA0747.1	SP8	11.82	0.898	hg38_knownG	610	621	+	aacacacccaca
MA0471.2	E2F6	11.80	0.900	hg38_knownG	432	444	+	ggagggggggaagg
MA1147.1	NR4A2::RXRA	11.79	0.864	hg38_knownG	309	323	-	gagttctttcacccc
MA1511.1	KLF10	11.79	0.921	hg38_knownG	827	837	+	agcacgccccc
MA0080.5	SPI1	11.78	0.813	hg38_knownG	1761	1780	-	gaaagggaggaaggaagga
MA1508.1	IKZF1	11.77	0.899	hg38_knownG	1747	1758	-	agaaaaggaaat
MA0813.1	TFAP2B(var.3)	11.75	0.895	hg38_knownG	1050	1062	-	tccccgtcgggcg
MA1638.1	HAND2	11.75	0.971	hg38_knownG	178	187	+	tacagatgtg
MA0747.1	SP8	11.74	0.897	hg38_knownG	638	649	+	ccccctccccct
MA0741.1	KLF16	11.72	0.903	hg38_knownG	417	427	-	tcccctccccc
MA1653.1	ZNF148	11.72	0.878	hg38_knownG	737	748	+	ttccccaccccc
MA0471.2	E2F6	11.70	0.898	hg38_knownG	662	674	+	cccggcgggagcg
MA1564.1	SP9	11.68	0.900	hg38_knownG	1725	1736	+	tccccgcctccc
MA1116.1	RBPJ	11.68	0.968	hg38_knownG	232	241	+	actgggaaac
MA1522.1	MAZ	11.68	0.925	hg38_knownG	1727	1737	+	cccgcctcccc
MA0813.1	TFAP2B(var.3)	11.67	0.894	hg38_knownG	832	844	-	agccccgggggggc
MA0813.1	TFAP2B(var.3)	11.66	0.894	hg38_knownG	832	844	+	gccccccggggct

MA0872.1	TFAP2A(var.3)	11.65	0.889	hg38_knownG	1255	1267	-	cccccgccgggca
MA1517.1	KLF6	11.65	0.931	hg38_knownG	826	836	+	aagcacgcccc
MA0471.2	E2F6	11.64	0.897	hg38_knownG	1679	1691	-	gttggagggaaaa
MA0598.3	EHF	11.63	0.867	hg38_knownG	1744	1758	+	tttatttccttttct
MA0750.2	ZBTB7A	11.54	0.891	hg38_knownG	1136	1148	-	ctccggaagctgc
MA0471.2	E2F6	11.53	0.895	hg38_knownG	772	784	-	gttggcgggagtg
MA0814.2	TFAP2C(var.2)	11.50	0.884	hg38_knownG	381	394	+	tcgcctgagccgcg
MA1515.1	KLF2	11.49	0.934	hg38_knownG	609	619	+	caacacaccca
MA0632.2	TCFL5	11.49	0.940	hg38_knownG	695	704	+	gcgcgcgcat
MA0673.1	NKX2-8	11.48	0.978	hg38_knownG	539	547	-	ccacttcag
MA0815.1	TFAP2C(var.3)	11.48	0.895	hg38_knownG	1364	1376	+	ggccctgggggc
MA0764.2	ETV4	11.47	0.946	hg38_knownG	1762	1771	-	gaaggaagga
MA1650.1	ZBTB14	11.47	0.892	hg38_knownG	1232	1243	+	ccccgcggacgt
MA0478.1	FOSL2	11.44	0.931	hg38_knownG	97	107	-	ggttgactcat
MA0506.1	NRF1	11.43	0.918	hg38_knownG	689	699	+	gcggaggcgcg
MA0099.3	FOS::JUN	11.43	0.919	hg38_knownG	97	106	+	atgagtcaac
MA0076.2	ELK4	11.43	0.918	hg38_knownG	1137	1147	+	cagcttccgga
MA1548.1	PLAGL2	11.41	0.927	hg38_knownG	1369	1378	-	cggcccccca
MA0657.1	KLF13	11.40	0.833	hg38_knownG	825	842	+	aaagcacgccccccgggg
MA0815.1	TFAP2C(var.3)	11.40	0.894	hg38_knownG	1364	1376	-	gccccccagggcc
MA0050.2	IRF1	11.40	0.814	hg38_knownG	439	459	-	ttttgctctttctttccttcc
MA0502.2	NFYB	11.39	0.886	hg38_knownG	90	101	-	ctcataggccaa
MA0162.4	EGR1	11.39	0.859	hg38_knownG	1425	1438	+	cgacgcgcaccccc
MA0632.2	TCFL5	11.38	0.939	hg38_knownG	683	692	-	ccgcgcgctc
MA0039.4	KLF4	11.38	0.904	hg38_knownG	989	1000	+	cccccctcccct
MA1104.2	GATA6	11.36	0.890	hg38_knownG	261	273	-	tgctcttatcaaa

[illegible]a
