# Supplementary material for: FGF18–FGFR2 signaling triggers the activation of c-Jun–YAP1 axis to promote carcinogenesis in a subgroup of gastric cancer patients and indicates translational potential
Source: Oncogene. 2020 Sep 15;39(43):6647–63. doi: 10.1038/s41388-020-01458-x (PMC7581496; doi:10.1038/s41388-020-01458-x)
Supplement: Supplementary file 6 — Supplementary Table S5 [file 41388_2020_1458_MOESM6_ESM.docx]

**Supplementary Table S5.** Primers sequences for qRT-PCR and ChIP-qPCR

| Primer Name | Sequence (5' to 3') |
| --- | --- |
| FGFR1-F | CCCGTAGCTCCATATTGGACA |
| FGFR1-R | TTTGCCATTTTTCAACCAGCG |
| FGFR2-F | CAGAGACCAACGTTCAAGCA |
| FGFR2-R | GAGGAAGGCATGGTTCGTAA |
| FGFR3-F | CTGAAAGACGATGCCACTGA |
| FGFR3-R | ACACCAGGTCCTTGAAGGTG |
| FGFR4-F | CCATAGGGACCCCTCGAATAG |
| FGFR4-R | CAGCGGAACTTGACGGTGT |
| JUN-F | TTCTATGACGATGCCCTCAACGC |
| JUN-R | GCTCTGTTTCAGGATCTTGGGGTTAC |
| AMOT F | ACCTCGTGAAGTCATCCTCCA |
| AMOT R | CCTCCGAATCTCGCCCTCTA |
| CCND1 F | GCTGCGAAGTGGAAACCATC |
| CCND1 R | CCTCCTTCTGCACACATTTGAA |
| CCND3 F | TACCCGCCATCCATGATCG |
| CCND3 R | AGGCAGTCCACTTCAGTGC |
| CCNA2 F | CGCTGGCGGTACTGAAGTC |
| CCNA2 R | GAGGAACGGTGACATGCTCAT |
| CDK6 F | AGTCTGATTACCTGCTCCGC |
| CDK6 R | TCCAGAATCATTGCACCTGAG |
| CTGF F | GGGCCTATTCTGTCACTTCG |
| CTGF R | ACGTGCACTGGTACTTGCAG |
| GLI2 F | CTGCCTCCGAGAAGCAAGAAG |
| GLI2 R | GCATGGAATGGTGGCAAGAG |
| ITGB2 F | TGAGAGGAACAGGAAGTGTCAGG |
| ITGB2 R | CACTCCTGAGAGAGGACGCAC |
| MYC F | CGTCCTCGGATTCTCTGCTC |
| MYC R | TGTTCCTCCTCAGAGTCGCT |
| PPP2R2B F | CCACACGGGAGAATTACTAGCG |
| PPP2R2B R | TGTATTCACCCCTACGATGAACC |
| TGFB1 F | GGCCAGATCCTGTCCAAGC |
| TGFB1 R | GTGGGTTTCCACCATTAGCAC |
| TP73 F | ACTAGCTGCGGAGCCTCT |
| TP73 R | AGATTGAACTGGGCCGTGG |
| YAP1 F | CAGCAACTGCAGATGGAGAA |
| YAP1 R | ACATCCCGGGAGAAGACACT |
| YAP1 binding site 1-1 F | CTCACTGCACGTCCCAAAC |
| YAP1 binding site 1-1 R | AGGTCCCAGTTCTGCCATTT |
| YAP1 binding site 1-2 F | TCTTTAATATTAGCATCAGGGGTAA |
| YAP1 binding site 1-2 R | AGACTGATGTTGCACAATCCT |
| YAP1 binding site 2-1 F | TGAATGGGCAAGGAGGTCAT |
| YAP1 binding site 2-1 R | CAACTATTGCAAAAACTGTATGGT |
| YAP1 binding site 2-2 F | AAGAAATGAATGGGCAAGGA |
| YAP1 binding site 2-2 R | TCTTTAATATTAGCATCAGGGGTAA |
